# An Internet Intervention to Improve Asthma Management: Rationale and Protocol of a Randomized Controlled Trial

**DOI:** 10.2196/resprot.2695

**Published:** 2013-08-13

**Authors:** Amaël Arguel, Annie YS Lau, Sarah Dennis, Siaw-Teng Liaw, Enrico Coiera

**Affiliations:** ^1^Centre for Health InformaticsAustralian Institute of Health InnovationUniversity of New South WalesSydneyAustralia; ^2^Centre for Primary Health Care and EquityUniversity of New South WalesSydneyAustralia; ^3^School of Public Health & Community MedicineUniversity of New South WalesSydneyAustralia; ^4^Academic General Practice UnitFairfield HospitalSydneyAustralia

**Keywords:** asthma management, Internet intervention, personalized health record, personally controlled health management system, eHealth, asthma action plan

## Abstract

**Background:**

Many studies have shown the effectiveness of self-management for patients with asthma. In particular, possession and use of a written asthma action plan provided by a doctor has shown to significantly improve patients’ asthma control. Yet, uptake of a written asthma action plan and preventative asthma management is low in the community, especially amongst adults.

**Objective:**

A Web-based personally controlled health management system (PCHMS) called Healthy.me will be evaluated in a 2010 CONSORT-compliant 2-group (static websites verse PCHMS) parallel randomized controlled trial (RCT) (allocation ratio 1:1).

**Methods:**

The PCHMS integrates an untethered personal health record with consumer care pathways and social forums. After eligibility assessment, a sample of 300 adult patients with moderate persistent asthma will be randomly assigned to one of these arms. After 12 months of using either Healthy.me or information websites (usual care arm), a post-study assessment will be conducted.

**Results:**

The primary outcome measure is possession of or revision of an asthma action plan during the study. Secondary outcome measures include: (1) adherence to the asthma action plan, (2) rate of planned and unplanned visits to healthcare providers for asthma issues, (3) usage patterns of Healthy.me and attrition rates, (4) asthma control and asthma exacerbation scores, and (5) impact of asthma on life and competing demands, and days lost from work.

**Conclusions:**

This RCT will provide insights into whether access to an online PCHMS will improve uptake of a written asthma action plan and preventative asthma actions.

**Trial Registration:**

Trial Registration: Australian New Zealand Clinical Trials Registry ACTRN12612000716864; https://www.anzctr.org.au/Trial/Registration/TrialReview.aspx?id=362714 (Archived by WebCite at http://www.webcitation.org/6IYBJGRnW).

## Introduction

### Background

Around 300 million people worldwide currently suffer from asthma [1]. The mortality caused by asthma is significant since a global estimation attributes to this disease about 1 in every 250 deaths [1]. Living with asthma is also difficult. People with asthma report worse health and quality of life than those without asthma [2,3].

Solutions have been developed to help people with asthma cope with their condition [4]. One of the key elements to optimal asthma management is to achieve good control of the disease. To achieve that, patients’ knowledge in recognizing asthma symptoms, identifying risk factors, using medication, and managing asthma exacerbations is important. Further, the relationship between patients with asthma and healthcare providers is crucial to develop an efficient asthma self-management plan [5].

One of the most useful tools in facilitating effective asthma self-management is the use of an individualized written asthma action plan, which describes different tailored steps of actions for the patient to follow according to their asthma severity. Written asthma action plans are claimed to be one of the most effective means for asthma self-management in several systematic reviews [6-8]. A Cochrane review by Gibson and colleagues concludes that use of a written asthma action plan, combined with regular visits to the doctor and education about asthma, leads to fewer visits to the emergency department, less hospital admissions, better lung function, and improvement on symptoms [6]. Online social networks and personal health management systems represent an innovative intervention to help patients engage with clinicians, health services, and self-management [9]. Despite strong evidence for the efficacy of written asthma action plans, they are widely under-used. Even after over 20 years of recommendation, only about one in 5 people with asthma have a written asthma action plan [10].

In Australia, the prevalence of asthma is particularly high and represents one of the highest in the world [2,3]. More than 1 in 10 adults and children is estimated to be suffering from asthma, representing more than 2 million Australians [2]. Although prevalence of asthma has declined over the last decade, the mortality caused by this disease remains significant with 411 people reported to have died from asthma in 2009 in Australia. According to a survey from the Australian Council of Asthma Monitoring in 2007 and 2008, only 21.3% of all-age patients with asthma are reported to possess a written asthma action plan [2,3]. This rate dropped to 14.4% when considering a subgroup of people aged 15 years and over [2].

There are a number of explanations for the under-usage of written asthma action plans by patients. Firstly, the diagnosis of asthma may not have been made in primary care where symptoms have been treated, if the disease is not labeled then patients may not be provided with long-term self-management tools [11]. When asthma is properly diagnosed, some health professionals provide patients with oral instruction instead of a formal written asthma action plan [12]. Others may consider asthma action plans as inefficient tools because of a lack of information and a difference of perception of asthma between patients and doctors [13,14]. From the patient’s point-of-view, asthma action plans can also be perceived as irrelevant or be under-used because of a passive attitude toward their asthma. This can prevent them from taking personal control of their asthma [15,16]. Finally, another reason could be that patients do not visit healthcare professionals to obtain or to update their asthma action plan, or do not ask for it when they see their general practitioner [15].

For asthma, Internet-based self-management appears to be a promising approach to improve control of this condition [17-21]. The aim of this study is to test a Web-based personally controlled health management system (PCHMS), in supporting consumers with asthma to encourage the uptake and use of a personal written asthma action plan, and to proactively seek self-management advice and schedule planned general practitioner (GP) visits before experiencing an asthma exacerbation. The PCHMS, called *Healthy.me,* has been previously tested with patients undergoing in-vitro fertilization (IVF) [22], in a randomized controlled trial to improve uptake of influenza vaccination [23,24], and amongst university students about help-seeking behaviors for physical and emotional well-being [25,26].

### Study Aims and Hypotheses

Specific hypotheses to be tested in this study are that:

1. Consumers using a Web-based PCHMS with interactive and social features are more likely to follow evidence-based guideline recommendations for asthma management, as measured by the rates of obtaining or updating a written asthma action plan with their GP and the usage rates with the asthma action plan, measured by questionnaires;
2. Use of a PCHMS will contribute to improved asthma control and reduced rates of asthma exacerbation compared to those using only static websites (usual care arm).

## Methods

### Study Design

A randomized controlled trial with a 2-group parallel design (with intervention allocation ratio 1:1) will be used to evaluate the efficacy of the Web-based PCHMS *Healthy.me*, reported in accordance to the 2010 CONSORT statements [27](ACTRN12612000716864). The PCHMS is not part of usual care but aims to improve usual care as measured by compliance to evidence-based guidelines.

- Participants randomized to the *intervention* group will have immediate access to an interactive version of *Healthy.me* with full PCHMS features as described later, from the date they are recruited.
- Participants randomized to the *control* group will receive access to a static webpage, without PCHMS features or any interactive component. This webpage will offer links to Australian information websites about asthma.

### Participants

Participants meeting eligibility criteria will be invited to use *Healthy.me* for 12 months. Participants will be assigned to intervention (access to *Healthy.me*) or control (access to static website) by random allocation generated by a computerized random-number generator [28]. Participants may be randomly selected to attend a 1-hour interview (or focus group) to discuss their asthma management and feedback at the end of the study. The participant inclusion eligibility criteria are as follows: (1) aged 18 or above, (2) living in Australia at the time of the study, (3) easy access to the Internet and email on a regular basis, (4) doctor diagnosis of asthma, and (5) adequate English reading and written ability. Participants currently enrolled in other trials of *Healthy.me* are excluded.

#### Recruitment Strategy

The recruitment of participants will be made possible with the assistance of Asthma Foundation Australia, the National Asthma Council Australia, and other consumer groups which have an online presence, to advertise our study using their existing participant engagement methods (ie, electronic newsletters and website). Because the study requires participants who are familiar with the use of the Internet, special emphasis will be given to Internet-based recruitment. Calls for research participants will be made on Google, Facebook, Twitter, and other online/social media on a regular basis, as well as on an online notice board (Gumtree Australia). It will also be possible that some participants will be recruited by others participants (snowballing sampling) via sharing in social networks, and thanks to the “invite a friend” feature they will be able to use on *Healthy.me* website.

Interested participants will be directed to a website with detailed information about the study. All consenting participants (control and intervention) will then be directed to a secure website to complete an eligibility screening survey.

#### Ethical Concerns and Consent

Ethics approval for this study has been obtained from the University of New South Wales (UNSW) Human Research Ethics Committee (approval no. HC12213), and from the National Research and Evaluation Ethics Committee of the Royal Australian College of General Practitioners (RACGP) (approval no. NREEC 12-005). Eligible participants will complete their written consent form online; the revocation of consent form is also available online.

### Intervention and Control

All participant volunteers responding to the invitation are required to register online by providing consent, completing a 3-minute eligibility questionnaire. Those eligible will then be invited to complete a 10-minute online pre-study survey and to watch a 3-minute online tutorial about *Healthy.me*. All eligible participants will also complete a 5-minute monthly survey during the study and a 10-minute post-study survey at 12 months. The control and intervention arms of the trial run concurrently, meaning that the randomly allocated participants of the trial will be exposed to the same environmental condition (eg, season), and the same background of public health campaigns.

#### Description of Intervention

*Healthy.me* consists of the following features:

1. *Personal Health Record (PHR)*. Allows for self-recording of medical test results and health measurements.
2. *Pillbox*. Allows for self-recording of current medications and medication adherence.
3. *Schedule, to-do list, and reminders*. An online schedule to self-record and keep track of health-related appointments, to-do items, which sends email reminders, and allows participants to book appointments with their health service providers.
4. *Team*. A feature that allows the self-recording of clinical and non-clinical personnel looking after one’s health.
5. *Journeys*. Consumer-specific care pathways that provide knowledge for health service engagement and self-management in an actionable way. These pathways describe the different stages in the management of health conditions that can be used to personalize other PHR sections in the system, and provides advice on what to expect and how to prepare for each stage (Participants in this study will have access to three asthma management journeys with content developed in collaboration with Asthma Foundation NSW, and adapted from its website [29]).
6. *Social communication spaces*. Support rich interaction across the continuum of care between participants and clinicians. The features include: (1) a poll system in which participants will be able to answer simple health-related questions and compare their response with other participants’ aggregated and de-identified responses; (2) ability to send and receive email messages with other participants on *Healthy.me*; (3) *diary* which offers a private place (by default but with the possibility to share with other participants) for participants to write down their thoughts and feelings; and (4) forums (moderated by a GP and the research team).
7. *Online appointment booking service.* This feature allows participants to be directly connected by telephone and at no charge with their health professional after clicking on a button. The 3 steps of the protocol are: First, participant clicks on a “book now” graphic button in *Healthy.me* and confirms his/her telephone number as well as health professional’s one; Second, participant and health professional receive an automatic ongoing call from the service, and Third, participant and health professional are connected each other and can book an appointment over the phone.

#### Intervention Group and Exposure

The period of access to *Healthy.me* will vary depending on the date of participant registration (from 9 to 12 months). During the study *Healthy.me* will provide participants in the intervention group with information and forward email reminders about asthma, indications for managing their asthma or other medical concerns, should they wish to use that service.

The intervention will not modify in any way the standard procedures of healthcare provision by GP clinics.

A pilot study has been conducted with 9 adults including 3 participants with asthma. Issues on system usability, journey content, surveys, study protocol, and advertisement material have been resolved before recruiting participants with asthma to participate in their normal setting.

#### Control

Participants in the control group will receive only a static webpage with links to external evidence-based materials written for consumers on asthma management available in Australia without the PCHMS features listed previously (ie, myDr.com.au, HealthInSite.gov.au, and asthmaaustralia.org.au), and delayed access to the full interactive version of *Healthy.me* by 12 months.

### Sample Size

A conservative estimate of at least 300 participants with 150 in each arm is needed to detect a 15% point difference in possession rate of a written asthma action plan between the control group (14.4%) and the intervention group (29.4%). This estimate is calculated at 5% level of significance, 80% power (2-sided test), with an anticipated participant dropout rate approximately 25%.

The effect size estimate is based on previous studies using *Healthy.me* assessing the efficacy of Internet-based interventions on the uptake of preventative health actions [23], and on studies with interventions promoting the use of personal asthma action plans [13]. The base rate is the percentage of adults (ie, 15 years and over) with current asthma in Australia and possessing a written asthma action plan, reported in the Australia Centre for Asthma Monitoring analysis of the Australian Bureau of Statistics National Health Survey 2007-2008, and cited in the Australia Centre for Asthma Monitoring 2011 report [2].

### Outcome Measures

The primary measure is the number of participants who have obtained, or have updated, their written asthma action plan with a GP within the 12 months of the study. The secondary outcomes concern actual use rate of the written asthma action plan, number of unplanned visit to healthcare for asthma, usage of *Healthy.me*, asthma symptoms, and competing demands on health and asthma. Please see Table 1 below.

**Table 1:** Summary of outcome measures.

Outcome measures		Measurement time points & methods		Time	
			Baseline	Monthly	Completion
**Primary outcome**					
	Number of participants with a written asthma action plan	Pre and post-study surveys	X		X
**Secondary outcomes**					
	Number of participants reported using their written asthma action plan, obtained, or updated during the study	Post-study survey	X		X
	Rate of planned (non-urgent) visits to a healthcare professional (eg, GP) for routine asthma management	Post-study survey			X
	Rate of unplanned visits to a GP, emergency department, caused by worsening asthma	Post-study survey			X
	Website usage patterns (number and timing of hits, duration of access, uptake of specific functions) of *Healthy.me*	*Healthy.me* system logs			X
	Technology acceptance of *Healthy.me*	Measured via the “Scales for Perceived Usefulness and Perceived Ease of Use” [30]			X
	Asthma control	Measured via “Asthma Control Questionnaire” [31]		X	
	Asthma exacerbations	Measured via the “Asthma Exacerbation Questionnaire” [32]		X	
	Number of days lost from work or school	Measured via an additional question after the “Asthma Control Questionnaire” [31]		X	
	Competing demands on health and asthma	Lists of life priorities and top health issues, monthly made by participants.		X	
					
					
					

### Data Collection

Firstly, self-reported responses are collected by use of Internet-based survey, accessed online, or sent to participants via email (Table 2). Surveys will be hosted by “KeySurvey”, an in-house survey infrastructure available at UNSW [33]. All completed responses will be stored securely in a server managed by UNSW. These surveys are:

Secondly, during the study, participants’ actions on the *Healthy.me* system will be unobtrusively and automatically logged.

Thirdly, a subset of participants (up to 10% of the sample) may be selected, according to their experiences and their patterns of behaviors using *Healthy.me*, for a post-study semi-structured interview/focus group, eliciting their feedback on *Healthy.me* and asthma self-management.

### Analysis Plan

Statistical significance is defined as a *P*-value of less than .05 (determined using a two-tailed test). Data will be collected by online survey software KeySurvey [33] and analyzed using IBM SPSS Statistics 19 [34].

#### Baseline Comparison

Comparisons of baseline variables between PCHMS group and control group will be conducted using visual inspection, to verify the absence of abnormal measures and data. An assessment of the homogeneity of the variances of distributions from the two groups will be statistically verified before carrying out inferential statistical analyses.

#### Primary Analysis

Differences in proportions of participants visiting their GP to obtain/update their written asthma action plan during the study will be compared between control and PCHMS groups. All intervention recipients who had the opportunity to use the PCHMS but did not do so will be included in the primary analysis (intention-to-treat principle with Last Observation Carried Forward (LOCF) imputation procedure for missing values [35,36]). Differences in participant proportions between control and PCHMS groups will be analyzed using χ^2^test or Student’s *t*-test. Proportions will be reported with 95% confidence intervals. Adjustments for baseline characteristics and possible confounders, such as age and other demographics [2,3], smoking status [3], and asthma severity [2], will be made through the use of sequential logistic regression [37]. All baseline characteristics, and factors that may affect the written asthma action plan possession rate collected at post study (eg, past possession of an asthma action plan) will be entered at step 1 of the regression; and group allocation (PCHMS vs. control) will be entered at step 2.

#### Secondary and Ancillary Analysis

Differences in proportions of participants between different groups (eg, control vs. PCHMS) will be examined using χ^2^test based on data collected from pre-, monthly, and post-intervention questionnaires, for each of the following activities experienced at least once during the study: (1) visited a GP (or a healthcare professional) for an unplanned asthma visit; (2) used medications or remedy; and (3) experienced performance impairment. Differences in average number of days of absence per participant and differences in score distributions from questionnaires will be compared between control and PCHMS groups using Student’s *t*-test or non-parametric statistics. Reasons for receiving (or not receiving) written asthma action plan will be reported using descriptive statistics. Attrition rate for the use of *Healthy.me* will be assessed with system logs. Technology acceptance of *Healthy.me* will be measured via the “Scales for Perceived Usefulness and Perceived Ease of Use” [30,38]. Participants’ satisfaction and utilization of Healthy.me will be reported. Cost effectiveness of health service utilization [39] (upon availability and feasibility) will also be considered as well as economic costs from days lost from work/school. If any post-hoc comparison would need to be performed between unplanned groups of participants, a Holm’s sequential Bonferroni procedure [40] will be used in order to control a familywise error rate *FWER* inferior or equal to level α = .05.

### Study Procedure

Table 3 below summarizes participant procedures in the study. The duration of the study is expected to be 12 months.

Email will be the primary channel to communicate with participants for study information and reminders about survey completion. From the time participants are recruited until study completion, all participants (control and intervention) will receive an email each month to complete a 5-minute survey about their health in the past month. At study completion, all participants will receive an email asking them to complete a post-study survey. In order to ensure the completeness of data collection, there will be 2 follow-up emails sent 5 days apart from each other to remind those who have not completed each survey.

**Table 2:** Surveys used in the study.

Survey	Purpose
Screening survey	Eliciting participants’ eligibility criteria.
Pre-trial survey	To obtain participants’ demographics and experiences of asthma management at study enrolment.
Monthly follow-up questions	To obtain participants’ self-reported symptoms of asthma (ie, asthma control and exacerbation), impact on work and study due to asthma symptoms, and competing demands and health throughout the study.
Post-trial survey (12 months after the beginning of the study)	To obtain participants’ state of asthma control and asthma management. For those who receive the PCHMS version of *Healthy.me*, their perceived usefulness of *Healthy.me* will also be assessed.

**Table 3:** Stages of study procedure.

Stage of study	Procedure
**Online registration**	
	Eligibility screening survey
	Participant registration, study consent and *Healthy.me* tutorial (self-completed online)
**Baseline data**	
	Pre-study survey (self-completed online)
**Participant follow-up procedures**	
	Monthly 5-minute surveys (self-completed online)
	Post-study survey (self-completed online)
	Patterns of *Healthy.me* use (computer logs and data entered by participants)

#### Randomization

After consent each participant is randomly allocated to the intervention or control group, stratified by gender and level of asthma severity (intermittent vs. persistent), according to a sequence generated by a computerized random-number generator [28] using permutated blocks of 2, 4, and 8. The randomization sequence generation, participant enrolment and group allocation processes in this study are computerized online and do not involve interference from the investigators.

#### Allocation Concealment and Assessment

Since *Healthy.me* is a behavioral intervention it is not possible to completely blind participants to the intervention. However, allocation of participants is automatically randomized, then coded within the Internet-based survey tool “KeySurvey”. The surveys are automatically sent by the system to participants, so investigators involved in the study are blinded to group allocation until completion of the quantitative analyses. The group allocation is revealed to participants only after they consent to participate in the study following completion of the pre-study questionnaire. To minimize contamination of control participants who might interact closely with participants who are part of the intervention group, participants in the intervention group are asked not to share their *Healthy.me* access details with other people.

## Results

The recruitment of participants is ongoing and first results are expected in early 2014. The data collection should be complete by mid-2014.

## Discussion

### Limitations

There are several potential limitations in this study. Firstly, the number of participants meeting inclusion criteria at study completion might be low because the study will focus recruitment only in Australia. The other inclusion criteria such as age, Internet access, and English speaking skills, might be also restrictive for recruiting participant in the targeted population.

Secondly, there is a possible high attrition rates*.* The study’s outcomes will rely on data from participants’ self-reports. Due to the long duration of the study, over 12 months, it is possible to observe a high rate of attrition from participants and thus, a significant loss of data. To limit this, all participants will be actively requested to complete questionnaires by receiving monthly reminders via emails. Furthermore, the questionnaires used on a monthly-basis will be easy and convenient complete (short questionnaires with an online completion using multiple-choice questions).

Thirdly, there may not be representative of consumers with asthma. The study may be more appealing to younger participants who are interested or literate in computers, the Internet, or asthma self-management topics. These participants may be more enthusiastic about health and the Internet than the general asthma population.

Fourthly, as this is a pragmatic trial of a multifaceted intervention in a complex environment, it is possible that baseline variables associated with participants might also influence the outcome. For example, having a prior history of obtaining a written asthma action plan may predict future planned visits to GP, independent of any additional intervention. We will identify potential baseline variables that might influence outcomes, including age and smoking status, and test for unequal variance in the distribution of these variables in the intervention and control populations.

### Concluding Remarks

Most of the features used in the study have been recently tested in a *Healthy.me* trial on help-seeking behaviors for physical and emotional well-being in a university student population [25]. The results of that study have shown that some bundles of features could lead to behavioral changes in university health service utilization and reported help-seeking for physical and/or emotional concerns. These results were consistent with theoretical expectations of behavioral changes generated from the use of features in *Healthy.me*. Particularly, the Health Belief Model (HBM) provides insights on how information may be transformed into action when informational cues in the environment are linked to action [41]. In the present study, we expect that the use of the online appointment booking service will be significantly associated with visits to health professionals, and consequently with a high rate in obtaining an asthma action plan. Another lever of behavioral change in accord with the principle of self-monitoring, may be operate thanks to features which encourage self-refection and self-awareness, such as the Personal Health Record, the Pillbox, the Diary, and the Poll [42]. In addition, the social and interactive features used in *Healthy.me* (Forums, Poll, Message) should play an important role in minimizing attrition and in promoting utilization of the platform amongst participants during the 12 months of the study [9].

Our design of the present randomized controlled trial (RCT) focuses on the comparison of outcomes that unequivocally reflect a change in consumer behavior, and takes into account the complexity of intervention. Results of this study will offer new insights about the utility of a Personally Controlled Health Management System (PCHMS) for consumer engagement with e-health services and self-management. Our findings will provide specific answers to whether using a Web-based PHCMS, containing information and self-management tools that facilitate consumers to engage with health services, will improve the uptake of preventive asthma management actions such as the possession and use of a written asthma action plan.

## Abbreviations

GP: general practitioner
HBM: Health Belief Model
IVF: in-vitro fertilization
LOCF: Last Observation Carried Forward
PCHMS: personally controlled health management system
RCT: randomized controlled trial
UNSW: University of New South Wales

## Multimedia Appendix 1

CONSORT-EHEALTH checklist V1.6.2 [43].
