# Supplementary material for: An Internet Intervention to Improve Asthma Management: Rationale and Protocol of a Randomized Controlled Trial
Source: JMIR Res Protoc. 2013 Aug 13;2(2):e28. doi: 10.2196/resprot.2695 (PMC3742402; doi:10.2196/resprot.2695)
Supplement: Supplementary file 1 [file resprot_v2i2e28_app1.pdf]

CONSORT-EHEALTH Checklist V1.6.2 Report

(based on CONSORT-EHEALTH V1.6), available at [http://tinyurl.com/consort-ehealth-v1-6].

2695

Date completed

8/8/2013 0:20:54

by

Amael Arguel

An Internet Intervention to Improve Asthma Management: Randomized Controlled Trial

TITLE

1a-i) Identify the mode of delivery in the title

"An Internet Intervention"

1a-ii) Non-web-based components or important co-interventions in title

1a-iii) Primary condition or target group in the title

"Asthma Management"

ABSTRACT

1b-i) Key features/functionalities/components of the intervention and comparator in the METHODS section of the ABSTRACT

"Methods: The PCHMS integrates an untethered personal health record with consumer care pathways and social forums. After eligibility assessment, a sample of 300 adult patients with moderate persistent asthma will be randomly assigned to one of these arms. After 12 months of using either Healthy.me or information websites (usual care arm), a post-study assessment will be conducted."

1b-ii) Level of human involvement in the METHODS section of the ABSTRACT

1b-iii) Open vs. closed, web-based (self-assessment) vs. face-to-face assessments in the METHODS section of the ABSTRACT

1b-iv) RESULTS section in abstract must contain use data

1b-v) CONCLUSIONS/DISCUSSION in abstract for negative trials

INTRODUCTION

2a-i) Problem and the type of system/solution

"From the patient's point-of-view, asthma action plans can also be perceived as irrelevant or be under-used because of a passive attitude toward their asthma. This can prevent them from taking personal control of their asthma [16,17]. Finally, another reason could be that patients do not visit healthcare professionals to obtain or to update their asthma action plan, or do not ask for it when they see their general practitioner [16]."

2a-ii) Scientific background, rationale: What is known about the (type of) system

"The PCHMS, called Healthy.me, has been previously tested with patients undergoing in-vitro fertilization (IVF) [23], in a randomized controlled trial to improve uptake of influenza vaccination [24,25], and amongst university students about help-seeking behaviors for physical and emotional well-being [26,27]."

METHODS

3a) CONSORT: Description of trial design (such as parallel, factorial) including allocation ratio

"For asthma, Internet-based self-management appears to be a promising approach to improve control of this condition [18-22]. The aim of this study is to test a Web-based personally controlled health management system (PCHMS), in supporting consumers with asthma to encourage the uptake and use of a personal written asthma action plan, and to proactively seek self-management advice and schedule planned general practitioner (GP) visits before experiencing an asthma exacerbation."

3b) CONSORT: Important changes to methods after trial commencement (such as eligibility criteria), with reasons

Article is about a protocol description

3b-i) Bug fixes, Downtimes, Content Changes

4a) CONSORT: Eligibility criteria for participants

"The participant inclusion eligibility criteria are as follows: (1) aged 18 or above, (2) living in Australia at the time of the study, (3) easy access to the Internet and email on a regular basis, (4) doctor diagnosis of asthma, and (5) adequate English reading and written ability. Participants currently enrolled in other trials of Healthy.me are excluded"

4a-i) Computer / Internet literacy

" easy access to the Internet and email on a regular basis"

4a-ii) Open vs. closed, web-based vs. face-to-face assessments:

"The recruitment of participants will be made possible with the assistance of Asthma Foundation Australia, the National Asthma Council Australia, and other consumer groups which have an online presence, to advertise our study using their existing participant engagement methods (ie, electronic newsletters and website). Because the study requires participants who are familiar with the use of the Internet, special emphasis will be given to Internet-based recruitment. Calls for research participants will be made on Google, Facebook, Twitter, and other online/social media on a regular basis, as well as on an online notice board (Gumtree Australia). It will also be possible that some participants will be recruited by others participants (snowballing sampling) via sharing in social networks, and thanks to the "invite a friend" feature they will be able to use on Healthy.me website."

4a-iii) Information giving during recruitment

"Interested participants will be directed to a website with detailed information about the study. All consenting participants (control and intervention) will then be directed to a secure website to complete an eligibility screening survey."

4b) CONSORT: Settings and locations where the data were collected

"self-reported responses are collected by use of Internet-based survey, accessed online, or sent to participants via email (Table 2). Surveys will be hosted by "KeySurvey", an in-house survey infrastructure available at UNSW [34]. All completed responses will be stored securely in a server managed by UNSW."

"during the study, participants' actions on the Healthy.me system will be unobtrusively and automatically logged"

"a subset of participants (up to 10% of the sample) may be selected, according to their experiences and their patterns of behaviors using Healthy.me, for a post-study semi-structured interview/focus group, eliciting their feedback on Healthy.me and asthma self-management"

4b-i) Report if outcomes were (self-)assessed through online questionnaires

"self-reported responses are collected by use of Internet-based survey, accessed online, or sent to participants via email (Table 2). Surveys will be hosted by “KeySurvey”, an in-house survey infrastructure available at UNSW [34]. All completed responses will be stored securely in a server managed by UNSW."

4b-ii) Report how institutional affiliations are displayed

5) CONSORT: Describe the interventions for each group with sufficient details to allow replication, including how and when they were actually administered

5-i) Mention names, credential, affiliations of the developers, sponsors, and owners

"The university and some of the researchers involved in this project could in the future benefit from any commercialization of Healthy.me or its technologies."

5-ii) Describe the history/development process

5-iii) Revisions and updating

5-iv) Quality assurance methods

5-v) Ensure replicability by publishing the source code, and/or providing screenshots/screen-capture video, and/or providing flowcharts of the algorithms used

5-vi) Digital preservation

5-vii) Access

"All participant volunteers responding to the invitation are required to register online by providing consent, completing a 3-minute eligibility questionnaire"

"The period of access to Healthy.me will vary depending on the date of participant registration (from 9 to 12 months)."

5-viii) Mode of delivery, features/functionalities/components of the intervention and comparator, and the theoretical framework

"Healthy.me consists of the following features:

1.Personal Health Record (PHR). Allows for self-recording of medical test results and health measurements.

2.Pillbox. Allows for self-recording of current medications and medication adherence.

3.Schedule, to-do list, and reminders. An online schedule to self-record and keep track of health-related appointments, to-do items, which sends email reminders, and allows participants to book appointments with their health service providers.

4.Team. A feature that allows the self-recording of clinical and non-clinical personnel looking after one’s health.

5.Journeys. Consumer-specific care pathways that provide knowledge for health service engagement and self-management in an actionable way. These pathways describe the different stages in the management of health conditions that can be used to personalize other PHR sections in the system, and provides advice on what to expect and how to prepare for each stage (Participants in this study will have access to three asthma management journeys with content developed in collaboration with Asthma Foundation NSW, and adapted from its website [30]).

6.Social communication spaces. Support rich interaction across the continuum of care between participants and clinicians. The features include: (1) a poll system in which participants will be able to answer simple health-related questions and compare their response with other participants’ aggregated and de-identified responses; (2) ability to send and receive email messages with other participants on Healthy.me; (3) diary which offers a private place (by default but with the possibility to share with other participants) for participants to write down their thoughts and feelings; and (4) forums (moderated by a GP and the research team).

7.Online appointment booking service. This feature allows participants to be directly connected by telephone and at no charge with their health professional after clicking on a button. The 3 steps of the protocol are: First, participant clicks on a “book now” graphic button in Healthy.me and confirms his/her telephone number as well as health professional’s one; Second, participant and health professional receive an automatic ongoing call from the service, and Third, participant and health professional are connected each other and can book an appointment over the phone."

5-ix) Describe use parameters

5-x) Clarify the level of human involvement

5-xi) Report any prompts/reminders used

"Email will be the primary channel to communicate with participants for study information and reminders about survey completion. From the time participants are recruited until study completion, all participants (control and intervention) will receive an email each month to complete a 5-minute survey about their health in the past month. At study completion, all participants will receive an email asking them to complete a post-study survey. In order to ensure the completeness of data collection, there will be 2 follow-up emails sent 5 days apart from each other to remind those who have not completed each survey."

5-xii) Describe any co-interventions (incl. training/support)

"The intervention will not modify in any way the standard procedures of healthcare provision by GP clinics."

6a) CONSORT: Completely defined pre-specified primary and secondary outcome measures, including how and when they were assessed

Table 1. Summary of outcome measures.
Outcome measuresMeasurement time points & methodsTime
BaselineMonthlyCompletion
Primary outcome
Number of participants with a written asthma action planPre and post-study surveysXX
Secondary outcomes
Number of participants reported using their written asthma action plan, obtained, or updated during the study Post-study surveyXX
Rate of planned (non-urgent) visits to a healthcare professional (eg, GP) for routine asthma managementPost-study surveyX
Rate of unplanned visits to a GP, emergency department, caused by worsening asthmaPost-study surveyX
Website usage patterns (number and timing of hits, duration of access, uptake of specific functions) of Healthy.meHealthy.me system logsX
Technology acceptance of Healthy.me
Measured via the “Scales for Perceived Usefulness and Perceived Ease of Use” [31]
X
Asthma controlMeasured via “Asthma Control Questionnaire” [32]
X
Asthma exacerbationsMeasured via the “Asthma Exacerbation Questionnaire” [33]
X
Number of days lost from work or schoolMeasured via an additional question after the “Asthma Control Questionnaire” [32]
X
Competing demands on health and asthmaLists of life priorities and top health issues, monthly made by participants.X

6a-i) Online questionnaires: describe if they were validated for online use and apply CHERRIES items to describe how the questionnaires were designed/deployed

6a-ii) Describe whether and how “use” (including intensity of use/dosage) was defined/measured/monitored

6a-iii) Describe whether, how, and when qualitative feedback from participants was obtained

6b) CONSORT: Any changes to trial outcomes after the trial commenced, with reasons
Not applicable: protocol description
7a) CONSORT: How sample size was determined
7a-i) Describe whether and how expected attrition was taken into account when calculating the sample size
"A conservative estimate of at least 300 participants with 150 in each arm is needed to detect a 15% point difference in possession rate of a written asthma action plan between the control group (14.4%) and the intervention group (29.4%). This estimate is calculated at 5% level of significance, 80% power (2-sided test), with an anticipated participant dropout rate approximately 25%.
The effect size estimate is based on previous studies using Healthy.me assessing the efficacy of Internet-based interventions on the uptake of preventative health actions [24], and on studies with interventions promoting the use of personal asthma action plans [14]. The base rate is the percentage of adults (ie, 15 years and over) with current asthma in Australia and possessing a written asthma action plan, reported in the Australia Centre for Asthma Monitoring analysis of the Australian Bureau of Statistics National Health Survey 2007-2008, and cited in the Australia Centre for Asthma Monitoring 2011 report [4]."
7b) CONSORT: When applicable, explanation of any interim analyses and stopping guidelines
not applicable
8a) CONSORT: Method used to generate the random allocation sequence
"After consent each participant is randomly allocated to the intervention or control group, stratified by gender and level of asthma severity (intermittent vs. persistent), according to a sequence generated by a computerized random-number generator [29] using permutated blocks of 2, 4, and 8. The randomization sequence generation, participant enrolment and group allocation processes in this study are computerized online and do not involve interference from the investigators."
8b) CONSORT: Type of randomisation; details of any restriction (such as blocking and block size)
After consent each participant is randomly allocated to the intervention or control group, stratified by gender and level of asthma severity (intermittent vs. persistent), according to a sequence generated by a computerized random-number generator [29] using permutated blocks of 2, 4, and 8. The randomization sequence generation, participant enrolment and group allocation processes in this study are computerized online and do not involve interference from the investigators.
9) CONSORT: Mechanism used to implement the random allocation sequence (such as sequentially numbered containers), describing any steps taken to conceal the sequence until interventions were assigned
After consent each participant is randomly allocated to the intervention or control group, stratified by gender and level of asthma severity (intermittent vs. persistent), according to a sequence generated by a computerized random-number generator [29] using permutated blocks of 2, 4, and 8. The randomization sequence generation, participant enrolment and group allocation processes in this study are computerized online and do not involve interference from the investigators.
10) CONSORT: Who generated the random allocation sequence, who enrolled participants, and who assigned participants to interventions
After consent each participant is randomly allocated to the intervention or control group, stratified by gender and level of asthma severity (intermittent vs. persistent), according to a sequence generated by a computerized random-number generator [29] using permutated blocks of 2, 4, and 8. The randomization sequence generation, participant enrolment and group allocation processes in this study are computerized online and do not involve interference from the investigators.
11a) CONSORT: Blinding - If done, who was blinded after assignment to interventions (for example, participants, care providers, those assessing outcomes) and how
11a-i) Specify who was blinded, and who wasn’t
"Since Healthy.me is a behavioral intervention it is not possible to completely blind participants to the intervention. However, allocation of participants is automatically randomized, then coded within the Internet-based survey tool “KeySurvey”. The surveys are automatically sent by the system to participants, so investigators involved in the study are blinded to group allocation until completion of the quantitative analyses. The group allocation is revealed to participants only after they consent to participate in the study following completion of the pre-study questionnaire. To minimize contamination of control participants who might interact closely with participants who are part of the intervention group, participants in the intervention group are asked not to share their Healthy.me access details with other people. "
11a-ii) Discuss e.g., whether participants knew which intervention was the “intervention of interest” and which one was the “comparator”

11b) CONSORT: If relevant, description of the similarity of interventions
not relevant for ehealth trials

12a) CONSORT: Statistical methods used to compare groups for primary and secondary outcomes
"Primary Analysis
Differences in proportions of participants visiting their GP to obtain/update their written asthma action plan during the study will be compared between control and PCHMS groups. All intervention recipients who had the opportunity to use the PCHMS but did not do so will be included in the primary analysis (intention-to-treat principle with Last Observation Carried Forward (LOCF) imputation procedure for missing values [36,37]). Differences in participant proportions between control and PCHMS groups will be analyzed using $\chi 2$ test or Student's t-test. Proportions will be reported with 95% confidence intervals. Adjustments for baseline characteristics and possible confounders, such as age and other demographics [4,7], smoking status [7], and asthma severity [4], will be made through the use of sequential logistic regression [38]. All baseline characteristics, and factors that may affect the written asthma action plan possession rate collected at post study (eg, past possession of an asthma action plan) will be entered at step 1 of the regression; and group allocation (PCHMS vs. control) will be entered at step 2.
Secondary and Ancillary Analysis
Differences in proportions of participants between different groups (eg, control vs. PCHMS) will be examined using $\chi 2$ test based on data collected from pre-, monthly, and post-intervention questionnaires, for each of the following activities experienced at least once during the study: (1) visited a GP (or a healthcare professional) for an unplanned asthma visit; (2) used medications or remedy; and (3) experienced performance impairment. Differences in average number of days of absence per participant and differences in score distributions from questionnaires will be compared between control and PCHMS groups using Student's t-test or non-parametric statistics. Reasons for receiving (or not receiving) written asthma action plan will be reported using descriptive statistics. Attrition rate for the use of Healthy.me will be assessed with system logs. Technology acceptance of Healthy.me will be measured via the "Scales for Perceived Usefulness and Perceived Ease of Use" "

12a-i) Imputation techniques to deal with attrition / missing values
"All intervention recipients who had the opportunity to use the PCHMS but did not do so will be included in the primary analysis (intention-to-treat principle with Last Observation Carried Forward (LOCF) imputation procedure for missing values [36,37])."

12b) CONSORT: Methods for additional analyses, such as subgroup analyses and adjusted analyses
"Comparisons of baseline variables between PCHMS group and control group will be conducted using visual inspection, to verify the absence of abnormal measures and data. An assessment of the homogeneity of the variances of distributions from the two groups will be statistically verified before carrying out inferential statistical analyses."

RESULTS

13a) CONSORT: For each group, the numbers of participants who were randomly assigned, received intended treatment, and were analysed for the primary outcome
Not applicable: Protocol article

13b) CONSORT: For each group, losses and exclusions after randomisation, together with reasons
Not applicable: Protocol article

13b-i) Attrition diagram

14a) CONSORT: Dates defining the periods of recruitment and follow-up
"The recruitment of participants is ongoing and first results are expected in early 2014. The data collection should be complete by mid-2014."

14a-i) Indicate if critical "secular events" fell into the study period

14b) CONSORT: Why the trial ended or was stopped (early)
Not applicable: Protocol article

15) CONSORT: A table showing baseline demographic and clinical characteristics for each group
Not applicable: Protocol article

15-i) Report demographics associated with digital divide issues
Not applicable: Protocol article

16a) CONSORT: For each group, number of participants (denominator) included in each analysis and whether the analysis was by original assigned groups

16-i) Report multiple "denominators" and provide definitions
Not applicable: Protocol article

16-ii) Primary analysis should be intent-to-treat

17a) CONSORT: For each primary and secondary outcome, results for each group, and the estimated effect size and its precision (such as 95% confidence interval)
Not applicable: Protocol article

17a-i) Presentation of process outcomes such as metrics of use and intensity of use

17b) CONSORT: For binary outcomes, presentation of both absolute and relative effect sizes is recommended
Not applicable: Protocol article

18) CONSORT: Results of any other analyses performed, including subgroup analyses and adjusted analyses, distinguishing pre-specified from exploratory
Not applicable: Protocol article

18-i) Subgroup analysis of comparing only users

19) CONSORT: All important harms or unintended effects in each group
Not applicable: Protocol article

19-i) Include privacy breaches, technical problems

19-ii) Include qualitative feedback from participants or observations from staff/researchers

DISCUSSION

20) CONSORT: Trial limitations, addressing sources of potential bias, imprecision, multiplicity of analyses

20-i) Typical limitations in ehealth trials

"There are several potential limitations in this study. Firstly, the number of participants meeting inclusion criteria at study completion might be low because the study will focus recruitment only in Australia. The other inclusion criteria such as age, Internet access, and English speaking skills, might be also restrictive for recruiting participant in the targeted population.
Secondly, there is a possible high attrition rates. The study's outcomes will rely on data from participants' self-reports. Due to the long duration of the study, over 12 months, it is possible to observe a high rate of attrition from participants and thus, a significant loss of data. To limit this, all participants will be actively requested to complete questionnaires by receiving monthly reminders via emails. Furthermore, the questionnaires used on a monthly-basis will be easy and convenient complete (short questionnaires with an online completion using multiple-choice questions).
Thirdly, there may not be representative of consumers with asthma. The study may be more appealing to younger participants who are interested or literate in computers, the Internet, or asthma self-management topics. These participants may be more enthusiastic about health and the Internet than the general asthma population.
Fourthly, as this is a pragmatic trial of a multifaceted intervention in a complex environment, it is possible that baseline variables associated with participants might also influence the outcome. For example, having a prior history of obtaining a written asthma action plan may predict future planned visits to GP, independent of any additional intervention. We will identify potential baseline variables that might influence outcomes, including age and smoking status, and test for unequal variance in the distribution of these variables in the intervention and control populations."

21) CONSORT: Generalisability (external validity, applicability) of the trial findings

21-i) Generalizability to other populations

21-ii) Discuss if there were elements in the RCT that would be different in a routine application setting

22) CONSORT: Interpretation consistent with results, balancing benefits and harms, and considering other relevant evidence

22-i) Restate study questions and summarize the answers suggested by the data, starting with primary outcomes and process outcomes (use)

Not applicable: Protocol article

22-ii) Highlight unanswered new questions, suggest future research

Not applicable: Protocol article

Other information

23) CONSORT: Registration number and name of trial registry

"Trial Registration: Australian New Zealand Clinical Trials Registry CTRN12612000716864 https://www.anzctr.org.au/Trial/Registration/TrialReview.aspx?id=362714 (Archived by WebCite at http://www.webcitation.org/6IYBJGRnW)"

24) CONSORT: Where the full trial protocol can be accessed, if available

Protocol article

25) CONSORT: Sources of funding and other support (such as supply of drugs), role of funders

"This research is supported by the National Health and Medical Research Council (NHMRC) Centre of Research Excellence in Informatics and E-Health (1032664)."

X26-i) Comment on ethics committee approval

x26-ii) Outline informed consent procedures

X26-iii) Safety and security procedures

X27-i) State the relation of the study team towards the system being evaluated
